# The interplay of stressful life events and coping skills on risk for suicidal behavior among youth students in contemporary China: a large scale cross-sectional study

**DOI:** 10.1186/s12888-015-0575-x

**Published:** 2015-07-31

**Authors:** Fang Tang, Fuzhong Xue, Ping Qin

**Affiliations:** 1 Department of Epidemiology and Biostatistics and Center for Suicide Prevention and Research, School of Public Health, Shandong University, Jinan, China; 2 Health Management Center, Qianfoshan Hospital Affiliated to Shandong University, Jinan, China; 3 National Centre for Suicide Research and Prevention, Institute of Clinical Medicine, University of Oslo, Sognsvannsveien 21, N-0372 Oslo, Norway

**Keywords:** Suicidal behavior, Stressful life event, Coping skills, Young people

## Abstract

**Background:**

Stressful life events are common among youth students and may induce psychological problems and even suicidal behaviors in those with poor coping skills. This study aims to assess the influence of stressful life events and coping skills on risk for suicidal behavior and to elucidate the underlying mechanism using a large sample of university students in China.

**Methods:**

5972 students, randomly selected from 6 universities, completed the questionnaire survey. Logistic regression analysis was performed to estimate the effect of stressful life events and coping skills on risk for suicidal behavior. Bayesian network was further adopted to probe their probabilistic relationships.

**Results:**

Of the 5972 students, 7.64 % reported the presence of suicidal behavior (attempt or ideation) within the past one year period. Stressful life events such as strong conflicts with classmates and a failure in study exam constituted strong risk factors for suicidal behavior. The influence of coping skills varied according to the strategies adapted toward problems with a high score of approach coping skills significantly associated with a reduced risk of suicidal behavior. The Bayesian network indicated that the probability of suicidal behavior associated with specific life events was to a large extent conditional on coping skills. For instance, a stressful experience of having strong conflicts with classmates could result in a probability of suicidal behavior of 21.25 % and 15.36 % respectively, for female and male students with the score of approach coping skills under the average.

**Conclusions:**

Stressful life events and deficient coping skills are strong risk factors for suicidal behavior among youth students. The results underscore the importance of prevention efforts to improve coping skills towards stressful life events.

## Background

Suicidal behavior in young people is highly associated with life stressors and also confers a substantial distress in their own right [1, 2]. A serious consideration of suicide or a non-fatal attempt of suicide can affect the quality of a person’s life, and serves as a powerful predictor of repetition of suicide attempt and even suicide completion [1, 3, 4]. Improvement in the ability to predict suicidal ideation or attempt through the continued identification of specific risk factors represents one of the most important directions in studying suicidal behavior among individuals of young ages [3].

Young people studying at universities and colleges often encounter various negative life events and stressors. Some students may not be able to reasonably analyze the importance and possible consequence of a perceived event, especially those with poor coping skills. Such instances, if occurred, would place them at risk to develop negative or depressed moods, leading to psychological problems and even suicidal behaviors in serious cases.

It has been reported that around 10 % of college students seriously considered to die from suicide and 1 to 3 % attempted suicide within the past school year [5–7]. Stressful life events, such as a failure in study exam, break-up of a love relationship, etc., are important contributors to suicidal behavior among the young students [2, 8–10]. At the same time, personal coping skills play an important role in the process of handling life stressors, in which approach coping is generally associated with better stressor resolutions whereas avoidance coping is associated with worse outcomes [11]. Several studies have consistently demonstrated a risk effect of avoidance coping but a protective effect of approach coping on the development of suicidal behavior among college students [11–13]. When an appropriate coping style is used, positive emotion could occur even when depression and distress are frequent [14]. A failure of effective coping with stress may then lead to psychological problems and even suicidal behaviors [11, 15]. Overall, there has been consensus that adequate coping in the developmental phase predicts good future outcomes, including higher levels of ego development and fewer behavioral problems [16], higher self-esteem [17], lower levels of psychological problems [18]. To better understand the complexity between stressful life events, coping skills and suicidality, rigorous research is essential to elucidate possible mechanisms underlying their relationships.

In this study, we want to use data from a large sample of undergraduate students from 6 universities in China to assess the influences of common stressful life events and personal coping skills on the risk for suicidal ideation or attempt. We also want to explore the mechanic role of coping skills on the relationship between life events and risk for suicidal behavior in this group of young people.

## Methods

### Selection of study subjects

Of 8 universities attached directly to the ministries of the P. R. China in Wuhan city, 6 universities agreed to join the survey for this study. A stratified cluster sampling method was used to randomly draw a 10 % sample of all undergraduate students in each university. The cluster for sampling was study class, organized by study specialty and school year with usually 50-120 students in each class. For each university, a spreadsheet listing study classes with information on specialty, school year and number of students was obtained from the university’s central academic administration office. Upon the list, study classes were drawn using random numbers generated by the random function of a calculator until the cumulative number of students reached the expected number, i.e., 10 % of all undergraduate students in the university. Classes of specialties in medicine and psychology were excluded from the selection in order to reduce possible selection bias (knowledge of exposure status influences the identification of diseased and non-diseased study subjects) and recall bias (knowledge of disease status influences the determination of exposure status). In case a selected class had more than 100 students, a sample of 100 students was then randomly drawn from this class. Otherwise, all students in the selected class were enrolled into the study. The rationale for restricting the number of participants to be selected from large clusters was to avoid the overweight of big classes so that the representativeness of the selected students in the university was ensured. With this sampling procedure, a total of 7220 university students from 93 classes were selected as the study population and 6099 attended questionnaire survey for data collection, corresponding to a response rate of 84.4 %. Follow-up of the 1121 students who did not attend the survey indicated that most of these students were out of the university campus for their internship during the period when the survey was conducted whilst a small number of students chose not to participate in the study. Each enrolled student was assigned with an encrypted code unique to their student identification for the survey.

The survey was conducted online via a website specifically designed for this study. Access to the online questionnaires was restricted to the students enrolled into the study, using the unique encrypted code as the password for login. All students were informed about the purpose of the study, the confidentiality of personal information and the principle of voluntary.

The survey started with an overall introduction on the research purposes, and then move onto online instructions for specific questionnaire sections. Two pilot studies were carried out to examine the suitability and understandability of the questionnaires and several pre-tests were conducted to test the functionality of the website. There was no report of technical problems during the final online survey collecting data for the study. 5972 students completed all question items relevant to the present study and their data were therefore included in the analyses.

This study was approved by the Ethics Committee of Huazhong Normal University where the original data were collected for their project. The informed consents were obtained from all students who participated the study.

### Measurements

Data on general characteristics such as sex, age and study specialty were collected from each student. Suicidal behavior, stressful life events, coping skills and psychopathologic features were assessed through self-designed question items or via standard questionnaires as described below.

#### Suicidal behavior

The dependent variable of study was suicidal behavior, including both suicidal ideation and suicide attempt within the past one year period. It was assessed with two question items: (1) “Did you ever seriously consider killing yourself in the past 12 months?”, and (2) “Did you ever try or attempt to kill yourself in the past 12 months”. Both items were answered on a 3-point rating (0 = ‘never’, 1 = ‘sometimes’, 2 = ‘frequently’). Since only a small amount of students reported of having a suicide attempt and most of these students also reported the presence of suicidal ideation, the two outcomes were therefore combined in the analyses. An answer of ‘1’ or ‘2’ to either of the two questions was then regarded as the presence of suicidal behavior.

#### Stressful life event (SLE)

To evaluate recent negative life events, a self-designed questionnaire was developed to assess frustrations in different domains that university students typically encounter during their university life. The students were asked if they had encountered the following eight frustrations within the past 12-month period: (1) a failure in study exam, (2) rupture of a love relationship, (3) strong conflicts with classmates, (4) financial problems, (5) bereavement from losing a family member, (6) suffering an acute illness, (7) severe illness of a close family member and (8) disciplinary punishment. The scoring method for each item was 1 for Yes and 0 for No.

#### Coping skills

The Coping Response Inventory, a 48-item instrument developed by Moos [19], was used to assess cognitive and behavioral responses the students used to cope with recent problems or stressful situations. The responses on this Likert-scaled instrument range from 0 (not at all) to 3 (fairly often) and are distributed into 8 subscales with 6 question items in each. 4 of the 8 subscales evaluate approach coping styles, i.e., Logical Analysis (LA), Positive Reappraisal (PR), Seeking Guidance and Support (SG) and Problem Solving (PS). The remaining four subscales evaluate avoidance coping styles, i.e., Cognitive Avoidance (CA), Acceptance or Resignation (AR), Seeking Alternative Rewards (SR) and Emotional Discharge (ED). The scoring was processed in accordance with the instruction by Moos (1993) [19]. Levels of both approach and avoidance coping scales were indicated by a value of 1 (T-score ≤ 45), 2 (T-score 46-54) or 3 (T-score ≥ 55), corresponding to the levels below the vaverage, average, and above the average, respectively. In the present study, the reliability of this instrument was promising with a Cronbach’s alpha of 0.92 for the overall and 0.91 and 0.86, respectively for the two dimensional measurements of approach coping skills and avoidance coping skills.

#### Psychopathology

Psychopathology was assessed using the Chinese version of the Symptom Checklist-90 Revised (SCL-90-R)) [20]. The SCL-90-R is a widely used instrument that measures participants’ self-reported psychopathologic features on nine subscales including somatization, obsessive-compulsiveness, interpersonal sensitivity, depression, anxiety, hostility, phobia, paranoid ideation, and psychoticism. Each question is rated on a five point Likert scale from 0 to 4 with a higher score indicating a lower status of psychological health (0 for no distress, 4 for extreme distress). In this study, the Cronbach’s alpha of this instrument was 0.95 and for the nine subscales the alpha ranged from 0.82 (paranoid ideation) to 0.92 (depression). The nine symptom dimensions were included in the analysis primarily for the purpose of adjustment. The average score of each symptom dimension was calculated to reflect the status of psychopathologic features, and a value of 2 was used as the cut point for being positive or negative to the symptoms.

### Statistical analysis

Descriptive analyses were performed to profile the distribution of variables of interest in the study population. Unconditional logistic regression analysis was performed to assess the impact of stressful life events and coping skills on risk for suicidal behavior using the statistical package of SAS, version 9.1. Participants with suicidal behavior were included as the cases while those without suicidal behavior were the controls. Crude odds ratios were derived from univariate regression analysis. Adjusted odds ratios were generated by multivariate regression model including all variables under study as well as the 9 dementional psychopathology of the subjects.

A Bayesian network was developed to probe the mechanic role of coping skills underlying the relationship between stressful life events and suicidal behavior using Hugin 7.0. Bayesian networks are acyclic directed graphs modeling probabilistic dependencies among variables [21, 22]. They can be used to quantify the relationship of variables of interest and predict the probability of an incident through a series of nodes linked by arcs (arrows). In the network, the nodes represent variables, and the arcs indicate relationships between/among the variables. In this study, the Bayesian network was constructed by the flowing procedures [21, 23]: (1) **Nodes selection**: the nodes, i.e., variables of interest, were selected according to the literature review, expert consultation and the results of regression analyses of the study data; (2) **Structure learning**: literature review, expert consultation and necessary path condition algorithm (NPC algorithm) were applied for structure learning in the construction of the Bayesian network; (3) **Parameter learning**: Expectation-maximization algorithm (EM algorithm) was used to determine the distribution of nodes; (4) **Assessment of the established Bayesian network**: The area under the Receiver Operating Curve (ROC) was applied as criteria. In the present study, the ROC is 0.95 for suicidal behavior, indicating that the established Bayesian network has high sensitivity and specificity. After the establishment of the Bayesian network model, network inference was then conducted upon the Bayes theorem to probe the probabilistic relationship between important stressful life events and suicidal behavior conditionally on sex of subjects and personal coping skills.

## Results

### Description of the study population

The study population comprised 3191 male and 2781 female students of 16 to 25 years old (mean age: 20.85, SD: 0.58). Stressful life events within the past 12 months were common among these students with financial problems being most common (41.64 %), followed by rupture of a love relationship (18.05 %), a failure in study exam (17.48 %), bereavement from losing a family member (15.97 %), severe illness of a close family (13.25 %), strong conflicts with classmates (10.92 %), suffering an acute illness (7.38 %), and disciplinary punishment being least common one (1.39 %).

Regarding coping skills assessed with the Coping Response Inventory (CRI), the score of approach coping was mainly concentrated in the level 2, i.e., the average level (46.13 %). The score of avoidance coping was mainly at the level 3, i.e., above the average (60.21 %).

### Prevalence of suicidal behavior

In the study population, 7.64 % of the university students reported the presence of suicidal behavior within the past 12 months, including 7.27 % having a serious suicidal ideation and 1.31 % having a suicide attempt. Specifically, 6.61 % of males and 8.81 % of females reported a presence of suicidal behavior, and the rate was significantly higher in female than male students (*χ*^2^ = 10.17, p = 0.001).

Compared with students reporting no stressful life event, suicidal behavior was significantly more common in students who had recently experienced a stressful life event of any types included in the study. In particular, students who received a disciplinary punishment and who had strong conflicts with classmates reported the highest rate of suicide behavior (18.07 % and 15.80 %, respectively). In the meantime, the presence of suicide behavior differed significantly by personal coping skills with a higher rate in students with deficient coping skills. For instance, suicidal behavior was present in 12.46 % of students with approach coping score below the average level against 5.03 % of peer students with approach coping score above the average level. Details are shown in Table 1.

**Table 1:** Distribution of suicidal behavior according to independent variables in the study population

Variable	N	Presence of suicidal behavior		*χ* ^2^	*p*
		Yes, n (%)	No, n (%)		
Gender					
Male	3191	211 (6.61)	2980 (93.39)	10.17	0.0014
Female	2781	245 (8.81)	2536 (91.19)		
Stressful life event					
Failure in study exam					
no	4928	343 (6.96)	4585 (93.04)	18.23	<0.0001
yes	1044	113 (10.82)	931 (89.18)		
Rupture of a love relationship					
no	4894	343 (6.96)	4585 (93.04)	18.23	<0.0001
yes	1078	113 (10.82)	931 (89.18)		
Strong conflicts with classmates					
No	5320	353 (6.64)	4967 (93.36)	69.13	<0.0001
yes	652	103 (15.80)	549 (84.20)		
Financial problems					
no	3485	228 (6.54)	3257 (93.46)	14.18	0.0002
yes	2487	228 (9.17)	2259 (90.83)		
Bereavement from losing a family					
No	5018	359 (7.15)	4659 (92.85)	10.32	0.0013
Yes	954	97 (10.17)	857 (89.83)		
Suffering an acute illness					
No	5531	391 (7.07)	5140 (92.93)	34.07	<0.0001
Yes	441	65 (14.74)	376 (85.26)		
Severe illness of a close family					
No	5180	374 (7.22)	4806 (92.78)	9.56	0.0020
Yes	792	82 (10.35)	710 (89.65)		
Disciplinary punishment					
No	5889	441 (7.49)	5448 (92.51)	13.00	0.0003
Yes	83	15 (18.07)	68 (81.93)		
CRI					
Approach coping skills					
Under the average	891	111 (12.46)	780 (87.54)	53.37	<0.0001
Average	2755	228 (8.28)	2527 (91.72)		
Above the average	2326	117 (5.03)	2209 (94.97)		
Avoidance coping skills					
Under the average	119	16 (13.45)	103 (86.55)	18.82	<0.0001
Average	2257	134 (5.94)	2123 (94.06)		
Above the average	3596	306 (8.51)	3290 (91.49)		

### Suicide risk associated with recent life stressors and coping skills

Logistic analyses of the data showed that risk of suicidal behavior was significantly associated with being female, with the exposure to a stressful life event of any type under study, and with the exhibition of deficient coping skills in the unadjusted model (Table 2). When gender, stressful life events, coping skills and psychopathologic features were considered simultaneously in the adjusted model, the event of having strong conflicts with classmates had the strongest effect on the risk of suicidal behavior. A failure in study exam, suffering an acute illness, and bereaved from losing a family had a modest effect. A high score of approach coping styles remained to be significantly protective against suicidal behavior; however, the negative effects of avoidance coping seen in the unadjusted model were attenuated into insignificant in the adjusted model.

**Table 2:** Risk for suicidal behavior associated with stressful life events and personal coping skills in the students

Variable	N (%)		Risk for suicidal behavior (95%CI)	
	Suicidal behavior	No suicidal behavior	Crude odds ratio^a^	Adjusted odds ratio^b^
Gender				
Male	211 (46.27)	2980 (54.02)	1 (reference)	1 (reference)
Female	245 (53.73)	2536 (45.98)	1.36 (1.13-1.65)^**^	1.51 (1.23-1.86)^**^
Stressful life event				
Failure in study exam				
No	343 (75.22)	4585 (83.12)	1 (reference)	1 (reference)
Yes	113 (24.78)	931 (16.88)	1.62 (1.30-2.03)^**^	1.36 (1.06-1.75)^*^
Rupture of a love relationship				
No	343 (75.22)	4551 (82.51)	1 (reference)	1 (reference)
Yes	113 (24.78)	965 (17.49)	1.55 (1.24-1.94)^**^	1.17 (0.91-1.49)
Strong conflicts with classmates				
No	353 (77.41)	4967 (90.05)	1 (reference)	1 (reference)
Yes	103 (22.59)	549 (9.95)	2.64 (2.08-3.34)^**^	2.01 (1.54-2.61)^**^
Financial problems				
No	228 (50.00)	3257 (59.05)	1 (reference)	1 (reference)
Yes	228 (50.00)	2259 (40.95)	1.44 (1.19-1.75)^**^	1.13 (0.92-1.39)
Bereavement from losing a family				
No	359 (78.73)	4659 (84.46)	1 (reference)	1 (reference)
Yes	97 (21.27)	857 (15.54)	1.47 (1.16-1.86)^**^	1.30 (1.01-1.68)^*^
Suffering an acute illness				
No	391 (85.75)	5140 (93.18)	1 (reference)	1 (reference)
Yes	65 (14.25)	376 (6.82)	2.27 (1.71-3.01)^**^	1.47 (1.06-2.05)^*^
Severe illness of a close family				
No	374 (82.02)	4806 (87.13)	1 (reference)	1 (reference)
Yes	82 (17.98)	710 (12.87)	1.48 (1.15-1.91)^**^	1.05 (0.79-1.40)
Disciplinary punishment				
No	441 (96.71)	5448 (98.77)	1 (reference)	1 (reference)
Yes	15 (3.29)	68 (1.23)	2.73 (1.55-4.81)^**^	0.91 (0.44-1.86)
CRI				
Approach coping skills				
Under the average	111 (24.34)	780 (14.14)	1 (reference)	1 (reference)
Average	228 (50.00)	2527 (45.81)	0.63 (0.50-0.81)^**^	0.56 (0.42-0.73)^**^
Above the average	117 (25.66)	2209 (40.05)	0.37 (0.28-0.49)^**^	0.30 (0.22-0.41)^**^
Avoidance coping skills				
Under the average	16 (3.51)	103 (1.87)	1 (reference)	1 (reference)
Average	134 (29.39)	2123 (38.49)	0.41 (0.23-0.71)^**^	0.71 (0.38-1.34)
Above the average	306 (67.11)	3290 (59.64)	0.60 (0.35-1.03)	1.28 (0.67-2.44)

^a^Crude odds ratios were derived from univariate logistic regression analyses; ^b^Adjusted odds ratios were adjusted for psychopathologic features and all variables in the table simultaneously; ^*^p < 0.05; ^**^p < 0.01

### Probability of suicidal behavior estimated upon Bayesian network

**Fig. 1:**
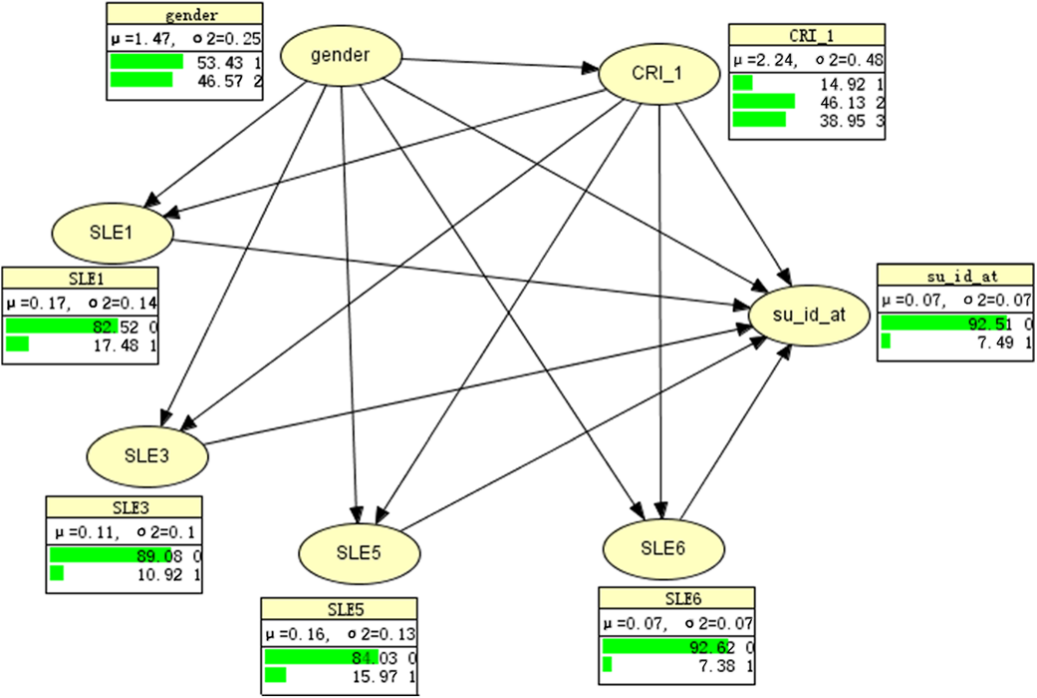
The proposed Bayesian network and the baseline distribution of variables of interest in the study population. CRI_1: approach coping skills; SLE1: failure in study exam; SLE3: strong conflicts with classmates; SLE5: bereavement from losing a family; SLE6: suffering an acute illness; su_id_at: suicidal behavior

Figure 1 presents the structure of Bayesian network established to probe the relationships of the selected variables (nodes) together with the baseline distribution of these variables in the study population. In each bin, the numeric column on the middle indicates the percentage (equivalent to the length of the horizontal green bar on the left) distribution of variable category specified by the numeric column on the right. For example, for the variable “SLE1”, the middle numeric column indicates that 17.48 % of the students included in this study experienced a failure in examination while 82.52 % did not.

According to this Bayesian network (the baseline model), we could assess how the exposure of a specific life event influences the probability of having suicidal behavior conditionally on specific gender and personal approach coping skills. Therefore, we calculated the conditional probability of suicidal behavior for nodes SLE1, SLE3, SLE5, SLE6, and CRI_1 for males and females in the pathway of Gender → CRI_1 → SLE (specific one) → Su-id-at (Table 3).

**Table 3:** Conditional probability for suicidal behavior, estimated upon the established Bayesian network

Pathway	Node			Probability of Suicidal behavior, in %
	Gender	CRI1	SLE1	
Gender → CRI1 → SLE1 → Su-id-at	1	1	1	10.59
	1	2	1	9.21
	1	3	1	6.72
	2	1	1	19.62
	2	2	1	15.28
	2	3	1	4.45
Pathway	Node			Probability of Suicidal behavior, in %
	Gender	CRI1	SLE3	
Gender → CRI1 → SLE3 → Su-id-at	1	1	1	15.36
	1	2	1	15.21
	1	3	1	8.76
	2	1	1	21.25
	2	2	1	20.61
	2	3	1	8.38
Pathway	Node			Probability of Suicidal behavior, in %
	Gender	CRI1	SLE5	
Gender → CRI1 → SLE5 → Su-id-at	1	1	1	11.02
	1	2	1	10.45
	1	3	1	5.36
	2	1	1	25.10
	2	2	1	9.95
	2	3	1	6.88
Pathway	Node			Probability of Suicidal behavior, in %
	Gender	CRI1	SLE6	
Gender → CRI1 → SLE6 → Su-id-at	1	1	1	10.19
	1	2	1	10.63
	1	3	1	11.99
	2	1	1	20.75
	2	2	1	16.71
	2	3	1	11.91

Gender (1 = male; 2 = female); CRI1: approach coping skills (1 = below the average; 2 = average level; 3 = above the average level); SLE1: failure in study exam (1 = yes); SLE3: strong conflicts with classmates (1 = yes); SLE5: bereavement from losing a close family member (1 = yes); SLE6: suffering an acute illness (1 = yes)

It is likely that, for both male and female students, the probability of suicidal behavior associated with the exposure of most specific life events differed conditionally on the level of approach coping skills. The conditional probability of suicidal behavior was most prominent for the stressor of bereavement from losing a family and having conflicts with classmates, respectively, for both female and male students, with a clear trend of decrease with increasing level of approach coping skills. A similar trend was also seen in both male and female students exposed to a failure in study exam, suggesting an important role of approach coping skills in the pathways from these life events to suicidal behavior. Taking the pathway Gender → CRI_1 → SLE3 → Su-id-at, for example, the exposure of having strong conflicts with classmates could result a probability of suicidal behavior of 21.25 %, 20.61 and 8.38 %, respectively, in female students with approach coping skills under the average level, on average and above the average level, respectively. The corresponding probability could be 15.36 %, 15.21 % and 8.76 %, respectively, for male students.

## Discussion

### Findings and possible explanations

In this study we have assessed the influences of stressful life events and coping skills on risk for suicidal behavior and probed their probabilistic relationships using a large sample of 5972 youth students from 6 universities in China. Three primary findings emerged: (1) 7.64 % of the students reported the presence of suicidal behavior (suicidal ideation or suicide attempt) within the past 12 months. (2) The risk of suicidal behavior was significantly associated with female gender, with the exposure of stressful life events and with the exhibition of deficient coping skills. (3) Based on the established Bayesian network, personal approach coping skills played an important role on the probabilistic relationship of suicidal behavior with exposures to life stressors, especially stressful events that have a prolonged effect on the students’ life.

Although any comparison with other study samples and populations should be cautiously interpreted because of the differences in sample sizes, methods and appraisal standards, the observed rate of suicidal behavior in our subjects (7.64 %, including 7.27 % for suicidal ideation and 1.31 % for suicide attempt) was in line with the reports from China [5, 6] and other countries [7, 24]. In the American National College Health Risk Behavior Survey, 9.5 % of students reported having seriously considered to die from suicide whilst 1.5 % having attempted suicide within the past school year [7]. Recent meta-analyses upon studies from China reported a pooled rate of 10.72 % (95 % CI: 8.41 % -13.28 %) for suicide ideation [5] and a pooled estimate of 2.9 % (95 % CI: 2.0 % -3.8 %) for suicide attempt [6] among the college students, although the reported rates varied greatly in individual studies [5, 6]. Also, the gender difference in suicidal behavior observed in our study population is highly concordant with the findings in previous studies from China [10, 25, 26] and western societies [2, 4, 24]. It is known that suicide mortality is generally higher in young males than females, but suicidal ideation and attempt are more common in female than male youth. Such phenomenon may be a consequence of sex-specific nature and nurture in the development of psychology [27], and seems to be universal and not influenced by culture and ethnic background.

Consistent with the literature [10, 13, 28–31], the present study supports the observation that risk of suicidal behavior is greater for individuals with, than those without, stressful, negative or potentially traumatic life experiences in their histories. It adds to the literature demonstrating the influences of common frustrations in different domains of university life as well as their relative importance on risk for suicidal behavior among the young people. In China, university students normally live in a university campus and share a dormitory room with other 3-7 classmate students. Because of the limited space for privacy, conflicts with classmates or roommates can arise and even lasts or escalates over time. Such experience could induce persisting distress in daily life and greatly affect their psychological well-being. This could largely explain our findings on the commonness of conflicts with classmates (10.92 %) in our study population, the high presence of suicidal behavior (15.80 %) among students with this stressor, and the particularly strong effect of this stressor on risk for suicidal behavior. At the same time, restricted school regulations in Chinese universities, high expectations from parents as well as rapid social and economic changes in the contemporary society of China may incur pressures and form chronic stressors on the students [13, 31]. This makes students particularly vulnerable to suicidal behavior under circumstances such as having a failure in exams or suffering an acute illness as demonstrated in our study. These findings indicate that, students with recent exposure to life stressors should be among the high priorities in mental health programs and in strategies for suicide prevention and intervention in university settings.

The role of coping skills has been extensively researched in understanding suicidal behavior among young people. A number of studies on college students have consistently indicated a direct effect of coping skills on suicidal behavior in this group of population [13, 15, 32]. Our results further demonstrate a protective effect of approach coping styles, i.e., the better the approach coping skills, the lower the risk for suicidal behavior. Hence, activities and training programs aiming to enhance personal approach coping skills is important in the efforts of suicide prevention in young people and would be particularly beneficiary to those recently exposed to negative life events and stresses.

To our awareness, the present study is the first to use the Bayesian network to probe the path-specific effect of important stressful life events and coping skills on suicidal behavior in young people. It is worth to note that, approach coping is problem-focused and reflects personal cognitive and behavioral efforts to master or resolve life stressors [19]. When young people meet a life stressor, they rely heavily on own approach coping skills to logically analyze the problem, to view it in a positive way, and to assertively deal with it. Failure to resolve an acute stressful event could lead to a prolonged exposure to stressful circumstances, increase reliance on avoidance coping, and induce psychological problems and even suicidal behavior. In line with these notions, our study demonstrates that the conditional probability of suicidal behavior associated with the exposure of a number of stressful life events increases with the decreasing score of approach coping skills of the students. On the other hand, our data also suggest that the role of coping skills underlying the link of a life event and suicidal behavior differs by type of the event. For instance, we did not see a clear trend of the conditional probability of suicidal behavior associated with the event of suffering an acute physical illness. It is therefore our interpretation that personal coping skills have a particularly prominent role on the relationship of suicidal behavior with stressful life events that have a prolonged effect and thus can affect the living atmosphere and routines of young people. These results are interesting and offer insights for our understanding on the role of coping skills on suicidal behavior in the young. We believe, efforts to strengthen young people’s reliance on cognitive and behavioral approach coping skills would have a significant effect on reducing suicidal behavior in this population, especially for individuals who often run into troublesome situations such as having conflicts with roommates or classmates, failing in study exams, etc.

### Strengths and limitations

The strengths of the present study include a large sample that was randomly drawn from all undergraduates in 6 universities, ensuring a good representativeness of students in these universities. The variables of study include a range of stressful life events and two dimensional coping styles, which covers various aspects important to university life and enables an insightful understanding of the interplay of these factors on risk for suicidal behavior. The study is, to our awareness, the first to apply a probabilistic graphical model to assess the predictive effect of specific life events on probability for suicidal behavior conditionally on coping skills of young students. The results are interesting and have strong clinical implications in suicide prevention.

The findings of this study should also be interpreted under several limitations. Firstly, our data were based on a cross-sectional questionnaire survey, which may affect the preciseness of collected information due to shortcomings commonly existing in this type of research, e.g., possible underreports, bias of recall, etc. Secondly, such a study design did not permit investigation of any causal relationship of the outcome with the exposures; neither made it possible to tell whether life events preceded suicidal behavior or the other way around. Moreover, cultural factors may have influenced the students’ willingness to report the presence of suicidal behavior [33], as in China suicidal behavior is usually regarded undesirable, especially among young people. Also, there are obvious differences in the living environment of Chinese students as compared with peer students in the Western societies. For instance, Chinese undergraduates usually live in a shared dormitory room located in a centralized campus whereas students in the West normally have own dormitories and live in different local areas. Therefore, some stressors assessed in the present study, e.g., conflicts with classmates, may not be a problem for young students in the Western society. Furthermore, suicide ideators and attempters are often seen as discrete groups [34], but the two outcomes were not analyzed separately in the present study to examine the differences. These limitations may impose a modest constraint on the interpretation of these findings, but they should not substantively undermine the internal validity of the study.

## Conclusions

The present study demonstrates that stressful life events and deficient coping skills are important risk factors for suicidal behavior in young students and that personal coping skills play a prominent role influencing the probability of having suicidal behavior among those recently exposed to stressful life events. These findings imply the importance of effort investment to students with poor coping skills towards stressful life events, and the need of such efforts to be included in programs of suicide prevention and mental health promotion in university settings.
